# An Efficient, Counter-Selection-Based Method for Prophage Curing in *Pseudomonas aeruginosa* Strains

**DOI:** 10.3390/v13020336

**Published:** 2021-02-21

**Authors:** Esther Shmidov, Itzhak Zander, Ilana Lebenthal-Loinger, Sarit Karako-Lampert, Sivan Shoshani, Ehud Banin

**Affiliations:** 1 The Mina and Everard Goodman Faculty of Life Sciences, Bar-Ilan University, Ramat-Gan 5290002, Israel; Estern95@gmail.com (E.S.); itzhak.zan@gmail.com (I.Z.); ilana.loinger@biu.ac.il (I.L.-L.); sivan.shoshani@biu.ac.il (S.S.); 2 The Institute of Nanotechnology and Advanced Materials, Bar-Ilan University, Ramat Gan 5290002, Israel; 3 Scientific Equipment Center, The Mina & Everard Goodman Faculty of Life Sciences Bar-Ilan University, Ramat Gan 5290002, Israel; sarit.lampert@gmail.com

**Keywords:** *Pseudomonas aeruginosa*, prophages, bacteriophages, curing, counter-selection, lysogen

## Abstract

Prophages are bacteriophages in the lysogenic state, where the viral genome is inserted within the bacterial chromosome. They contribute to strain genetic variability and can influence bacterial phenotypes. Prophages are highly abundant among the strains of the opportunistic pathogen *Pseudomonas aeruginosa* and were shown to confer specific traits that can promote strain pathogenicity. The main difficulty of studying those regions is the lack of a simple prophage-curing method for *P. aeruginosa* strains. In this study, we developed a novel, targeted-curing approach for prophages in *P. aeruginosa*. In the first step, we tagged the prophage for curing with an ampicillin resistance cassette (*ampR*) and further used this strain for the *sacB* counter-selection marker’s temporal insertion into the prophage region. The sucrose counter-selection resulted in different variants when the prophage-cured mutant is the sole variant that lost the *ampR* cassette. Next, we validated the targeted-curing with local PCR amplification and Whole Genome Sequencing. The application of the strategy resulted in high efficiency both for curing the Pf4 prophage of the laboratory wild-type (WT) strain PAO1 and for PR2 prophage from the clinical, hard to genetically manipulate, 39016 strain. We believe this method can support the research and growing interest in prophage biology in *P. aeruginosa* as well as additional Gram-negative bacteria.

## 1. Introduction

*Pseudomonas aeruginosa* is a Gram-negative, rod-shaped bacterium with a single flagellum. It is an opportunistic pathogen of plants, nematodes, insects, animals, and humans [1]. It can cause a wide range of acute and chronic infections, such as in severe wounds or burns, and chronic lung infections in cystic fibrosis (CF) patients [2,3]. This is enhanced by the bacterium’s low susceptibility to various antimicrobial substances, making most of the infections difficult to treat and life threatening [4,5]. The genetic variability among *P. aeruginosa* isolates is relatively low; different *P. aeruginosa* strains share a conserved “core genome” and differ in the variable “accessory segments” [6]. The accessory genome in *P. aeruginosa* consists mainly of genomic islands and mobile genetic elements (MGEs) such as plasmids, transposons, and prophages-like elements (temperate bacteriophages).

Bacteriophages (phages) are viruses that infect bacteria. They are highly abundant, rapidly spreading, and very diverse biological entities. Phage infections divide into productive lytic or lysogenic (temperate), depending on several factors such as host genetics, phage genetics, environmental conditions, and phage concentration [7]. In the lytic life cycle, phage infection is usually followed by intensive viral DNA replication, eventually leading to a high rate of new phage production and bacterial host cell lysis. In the lysogenic life cycle, the viral genome is integrated into the bacterial genome as a prophage and further replicates throughout bacterial cell division. A temperate phage can be triggered into the lytic cycle (excision event) spontaneously at a low frequency or by an external stressor such as bacterial DNA damage and physical or chemical factors [7]. The integration of a lysogenic phage into the bacterial genome can influence bacterial phenotypes by providing new traits such as antibiotic resistance, metabolic factors, as well as immunity against specific viral re-infections [7,8]. In some pathogenic bacterial species, prophages encode virulence factors and mediate bacterial adaptation in ecological niches [9]. As mobile elements, prophages are an essential tool for horizontal gene transfer between bacteria and, among other factors, contribute to strain genetic variability.

Most *P. aeruginosa* strains were identified to contain at least one prophage-like element; some are poly-lysogens, harboring several prophages in their genome [10,11]. Lysogenic phages in *P. aeruginosa* have been shown to confer selective beneficial traits, such as O antigen conversion, biofilm development, and virulence [12,13,14].

The PAO1 laboratory strain contains Pf1-like filamentous phage (Pf4) as a prophage that plays a role in small colony variant (SCV) formation during *P. aeruginosa* biofilm development and has a symbiotic role in bacterial biofilm formation [15,16]. Moreover, 39016 is a clinical isolate of *P. aeruginosa* that phenotypically differs from the laboratory wild-type (WT) strain PAO1; it exhibits a slower growth rate, reduced motility, and higher biofilm production levels [17]. The prophage-region screening of the published genome of the 39016 strain revealed that it contains five different prophage-like regions in its genome, and four of them are predicted to be intact and able to create infectious virions. As 39016 and PAO1 share the high conserved "core genome" and considering the phenotypic variation among these two strains, the prophage regions of 39016 are likely to influence the bacterial physiology and virulence.

A comparison of isogenic strains that differ only in their prophage region can contribute to understanding the biological role of a specific phage to the observed traits, the overall prophages contribution, and the cross-regulation between the prophages in poly-lysogenic strains. The main difficulty for this type of study is the lack of a simple and efficient experimental method for curing prophages in *P. aeruginosa* strains.

Previously described prophage curing methods, both for Gram-negative and Gram-positive bacteria, mostly relied on promoting the phage loss with the use of DNA-damaging reagents and the activation of the SOS-response and thus potentially causing a variety of genomic mutations in the bacterial genome, which might result in further variation of the phenotypes [18,19]. Here, we introduce a simple, non-SOS based, highly efficient prophage curing method in *P. aeruginosa*, using the Gram-negative popular counter-selection marker *sacB* [20]. We applied the prophage curing method on Pf4 from the WT strain PAO1 and a prophage from the clinical isolate 39016, herein termed PR2. The method can theoretically be applied for all the Gram-negative bacteria containing an intact, excisable prophage in their genome, allowing a better investigation of the prophages and their contribution to bacterial physiology and virulence.

## 2. Materials and Methods

### 2.1. Bacterial Strains, Plasmids and Growth Media

The bacterial strains and plasmids used in this study are listed in Table 1. Primers are listed in Supplementary Table S1. All strains were grown on LB (Luria-Bertani broth, Difco, Sparks, MD, USA) at 37 °C unless otherwise specified. For the insertion processes, the following media were used: Vogel Bonner Minimal Medium (VBMM) [21], *Pseudomonas* Isolation Agar (PIA, Difco, Sparks, MD, USA), and No salt Luria-Bertani (NSLB) + 10% sucrose. For the plasmid curing assay and DH5α heat shock, BHI (Brain Heart Infusion broth, Difco, Sparks, MD, USA) media was used. Antibiotic concentrations used in this study were 300 μg/mL carbenicillin (Crb) and 50 μg/mL gentamicin (Gm) for *P. aeruginosa* and 100 μg/mL ampicillin (Amp) and 30 μg/mL Gm for *Escherichia coli*.

### 2.2. DNA Manipulation and Plasmid Construction

Genomic extraction was performed using the DNeasy Blood & Cell Culture DNA Kit (Qiagen, Hilden, Germany). For DNA fragment amplification, Phusion High-Fidelity DNA Polymerase (Thermo) was used. DNA fragments upstream and downstream to the deletion/insertion region were amplified from genomic DNA using PCR. The amplified inserts were cleaned using NucleoSpin Gel and PCR Clean-up (MACHEREY-NAGEL). *ampR* cassette was amplified from the plasmid pUCP18-Ap using PCR (primers ampR_F and ampR_R). The segments were inserted into pEX18Gm-GW plasmid by recombination (Gateway recombination system and BP Clonase enzyme, Invitrogen, Carlsbad, CA, USA). The DNA Polymerase ReddyMix PCR Kit and M13_F and M13_R universal primers were used to verify successful plasmid transformations. The plasmids were extracted with QIAprep spin miniprep kit (QIAGEN, Hilden, Germany) and sequenced. All of these processes were carried out according to the manufacturer’s instructions.

### 2.3. Insertion of ampR into Phage Region

The PAO1/Pf4_ampR (Δ*pfiT*) and 39016/PR2_ampR strains construction was carried out as previously described [21] with minor changes using the *ampR* cassette. In PR2 prophage, the *ampR* cassette was inserted instead of the PA39016_000100015 gene. For Pf4 phage curing, the *ampR* gene was inserted inside the Pf4 locus at ORF PA0729 [23].

### 2.4. Prophage Curing and Selection for Phage-Cured Mutants

The *ampR*-containing strains were further inserted with *sacB* into the phage region, as previously described with some process adaptation changes [21]. Briefly, merodiploids with the pDONER/Pf4_SacB or the pDONER/PR2_SacB plasmid undergo site-specific integration into the Pf4 and PR2, respectively, by vector-encoded homologous sequences of prophage region. The merodiploids were streaked on NSLB plates containing 10% sucrose and were incubated for 24 h at 30 °C. Single colonies isolated on the sucrose agar were taken and transferred to PIA, LB Gm, and LB Crb plates and incubated overnight. Positive *P. aeruginosa* colonies that contained the desired deletion grew on PIA plates, but neither on LB Gm nor on LB Crb containing plates. The mutants were verified for complete loss of the phage by PCR for prophage genes and the intact bacterial attachment site *attB*.

### 2.5. Plasmid Curing

For recombinant plasmid curing, the 39016 ΔPR2/Gm^r^ culture was incubated overnight in BHI at 42 °C with shaking and then streaked overnight on LB plate. Single colonies isolated on LB plates were then transferred to the LB and LB Gm plates for Gm-sensitive variant identification.

### 2.6. Phage Extraction

LB (2 mL) was inoculated with bacterial strain and incubated overnight. The next day, the bacteria were diluted 1:50 with medium to a final volume of 4 mL and incubated for 3 h. Then, 1 mL of bacteria was centrifuged at 14,000× *g* for 2 min, and 900 µL of the supernatant was filtered using a 0.45 µm filter (Whatman, Maidstone, Kent, UK).

### 2.7. Plaque Assay

LB (2 mL) was inoculated with recipient strain and incubated overnight with shaking. The next day, bacteria were diluted 1:50 with medium to a final volume of 2 mL and incubated with shaking for 2 h. Further, 100 µL of the bacteria was transferred into 5 mL heated (50 °C) LB with 0.5% agar, and then gently mixed and poured onto the surface of a 1.5% LB-agar plate. Serial dilutions (1:10) of the extracted phage stock were made, droplets of 2 µL were spotted on to the top-agar layer. The plate was incubated overnight until plaques were formed.

### 2.8. Whole-Genome Sequencing

DNA was extracted from cell pellets using the DNeasy Blood & Tissue Kit (Qiagen), and DNA quality was evaluated by gel electrophoresis. The library was constructed with NEBNext Ultra II FS DNA Library Prep Kit for Illumina (NEB, UK) according to the manufacturer’s instructions using 500 ng as the starting material. DNA was fragmented 20 min with three cycles of PCR. The final quality was evaluated by TapeStation High Sensitivity D1000 Assay (Agilent Technologies, CA, USA). Sequencing was performed based on Qubit values and loaded onto an Illumina MiSeq using the MiSeq Micro 150× 2 kit (Illumina, CA, USA).

### 2.9. Genome Assembly and Sequence Analysis

The MiSeq sequencing resulted in 1.13–2.87 million paired-end reads per strain. FastQC (v0.11.2) (https://www.bioinformatics.babraham.ac.uk/projects/fastqc(accessed on 11 February 2021)) was used to assess the quality of the raw reads. For each strain de novo assembly was performed by SPAdes (v3.13.0) [26] with parameters -k 21,33,55,77,99,127 --careful. QUAST (v5.0.2) [27] was used for quality assessment of the genome assemblies. Contigs with a minimal length of 500 bases from the assemblies of *P. aeruginosa* 39016 (WT and mutant) and *P. aeruginosa* PAO1 (WT and mutant) were reordered according to reference genomes (NCBI Reference Sequence NZ_CM001020.1 and NC_002516.2, respectively) using Mauve aligner [28,29]. Regions of prophage insertion were determined using PHASTER [30]. BLAST Ring Image Generator (BRIG) (https://sourceforge.net/projects/brig/ (accessed on 11 February 2021)) [31], and Mauve [28] were used to compare the WT and mutant to the reference genome.

## 3. Results

### 3.1. The Targeted Curing Principle

**Figure 1:**
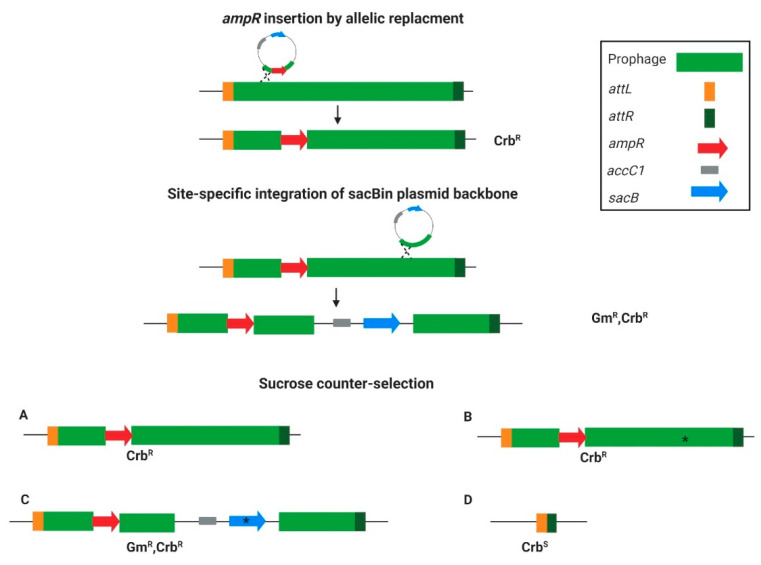
The targeted curing method principle. The prophage is bordered by the right attachment site *attR* (dark green) and the left attachment site *attL* (orange). The first step is *ampR* (red) insertion for prophage tagging. The second step is the integration of the plasmid backbone, containing *accC1* (gray) and *sacB* (light blue) into the prophage region via homologous recombination. Next, the temporal tagged prophage with the integrated *sacB* undergoes counter-selection by growing on the sucrose-containing growth medium. The counter-selection outcome can be either (**A**) unchanged tagged prophage, (**B**) integration site deletion (marked with an asterisk), (**C**) *sacB* mutated variant (asterisk), or (**D**) prophage curing. Notably, only option (**D**) would be Crb sensitive.

The overall principle of the curing strategy is presented in Figure 1. The first step was to tag the targeted prophage with an *ampR* cassette insertion into the phage region using an allelic replacement technique. After creating the ampicillin-resistant strain, it was further used for *sacB* insertion by utilizing the site-specific integration step of the gentamicin resistance marker (*aacC1*) and *sacB* containing plasmid backbone in the allelic replacement method. Homologous regions were used to direct the backbone integration into the prophage, and in this way, a temperate *ampR/sacB/aacC1* containing-form of the prophage was created (see scheme in Figure 1). To push toward either insertion or the desired prophage curing, the merodiploids were streaked on sucrose plates. The sucrose counter-selection resulted in either no change in the *ampR*-inserted prophage (Figure 1A), integration-site deletion mutants (Figure 1B), *sacB* mutants that survived the sucrose selection (Figure 1C), or the whole prophage-cured variant (Figure 1D). Out of all these options, the prophage-cured mutant is the sole Crb-sensitive variant (Figure 1). Such a system makes it possible to select for rare colonies that lost the *ampR*-inserted prophage. The separation of the two steps was to assure insertion distance between the counter-selection and the positive-selection markers to avoid false-positive results in variants that lost all the markers but not the whole prophage.

### 3.2. Pf4 Phage of PAO1 Curing

The curing of Pf4 prophage of the PAO1 strain was carried out to demonstrate the efficiency of the targeted curing method.

First, PAO1/Pf4_*ampR* (Δ*pfiT*) mutant was created by inserting the *ampR* cassette instead of the PAO729 gene, which encodes for the PfiT toxin protein from the toxin–antitoxin (TA) *pfiT/pfiA* system. The insertion did not alter the bacterial growth compared to WT PAO1, and the strain still showed spontaneous phage loss [23].

To further integrate the *sacB*-containing plasmid backbone, the upstream and downstream homologous sequences of the PA0725 gene were used to direct the insertion into the prophage and create the transient *ampR/sacB/accC1* containing Pf4-containing prophage. The counter-selection on sucrose-containing medium resulted in a high yield; 100% of the analyzed colonies (40/40) were both Crb- and Gm-sensitive, indicating a potential phage loss.

**Figure 2:**
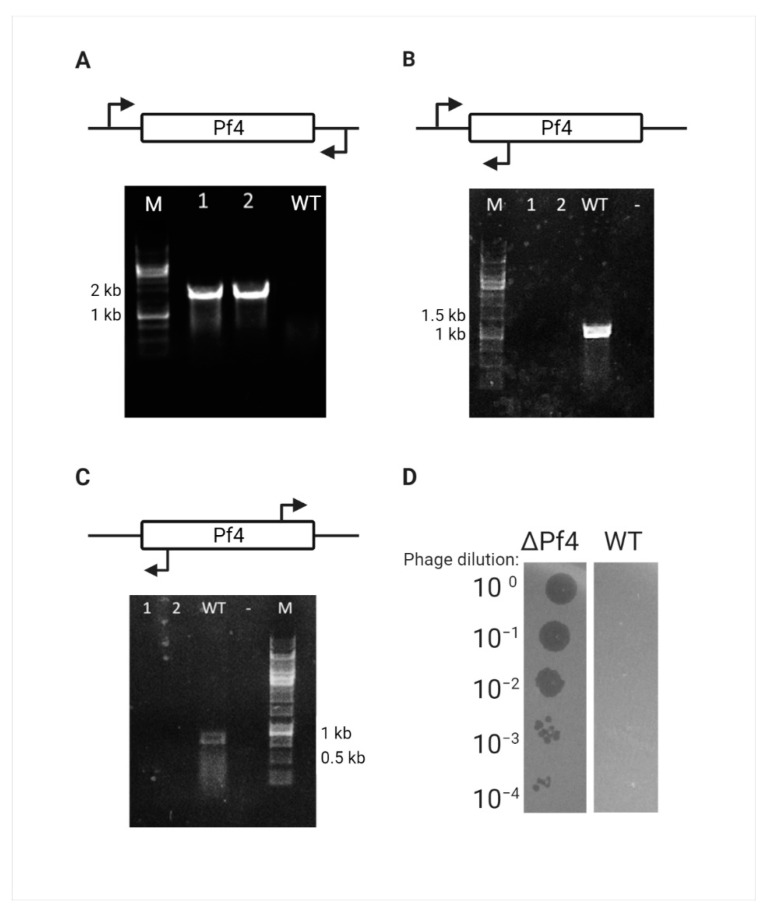
Pf4 curing verification. (A-C) Curing verification by PCR, Lanes 1–2 represent the DNA of randomly picked Crb-sensitive colonies, and lane 3 represents the WT PAO1 for control. (**A**) Amplification of 1800 bp around the Pf4 *attB* site. (**B**) Amplification of 1000 bp around the Pf4 *attR* site that can only be amplified in the integrated form. (**C**) Amplification of 750 bp around the Pf4 *attP* site that can only be amplified in the RF form. (**D**) Curing verification by plaque assay, serial dilutions of Pf4 phage are extracted from WT PAO1 strain used to infect WT PAO1 and ΔPf4 strain.

Complete loss of the Pf4 prophage and the circular replicative form (RF) DNA was confirmed for this mutant by PCR amplification around the *attB* site of Pf4, the *attR* site for the integrated form, and the phage attachment site (*attP*) for the RF form of Pf4 phage (Figure 2A–C). One such mutant was randomly chosen and designated as ΔPf4 strain. As the WT PAO1 strain exhibits immunity properties against Pf4 re-infection, the ΔPf4 strain was tested for immunity loss. The cured strain infection with Pf4 phages, extracted from WT PAO1, resulted in plaque formation and bacterial susceptibility, unlike the WT strain (Figure 2D).

**Figure 3:**
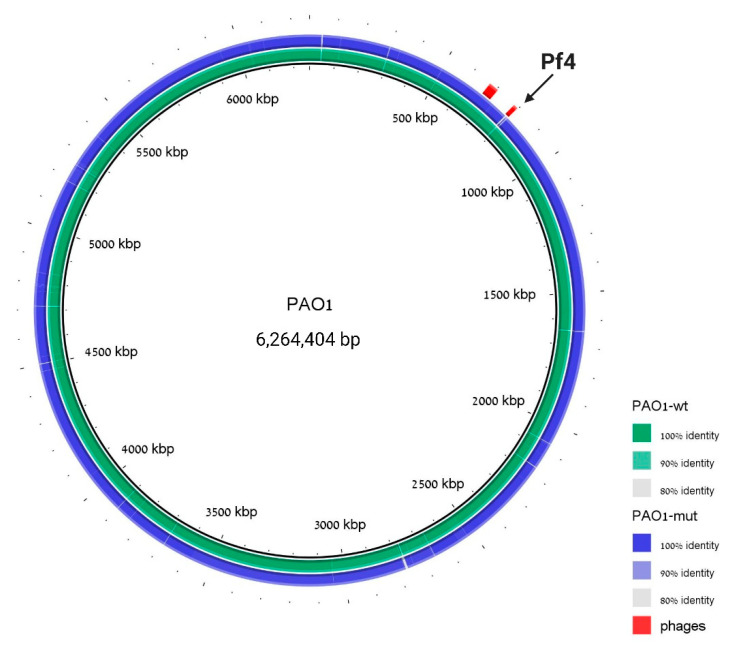
The ΔPf4 strain differs from WT in the Pf4 region. Genomic alignment with PAO1 strain is used as a reference, PAO1 WT sequence assembly is indicated in green, and ΔPf4 strain sequence assembly is indicated in blue. Prophage regions are labeled in red; coordinates are taken from PHASTER analysis to the reference PAO1 strain. The cured region is marked with an arrow. We note that there is a difference between our laboratory WT strain and the reference strain around region 2800 bp, but this difference is identical in the Pf4-mutant strain.

To assure the genome integrity of this strain and to prove once more the phage loss, whole-genome sequencing was performed to the cured strain, and the resulting assembly was aligned to the PAO1 WT sequence. The genome analysis revealed that the WT strain assembly covers 99.284% of the reference genome and that besides the Pf4 region, no additional significant insertions or deletions were detected in the mutant strain (Figure 3).

### 3.3. PR2 Phage of 39016 Curing

Clinical isolates of *P. aeruginosa* are less attenuated than the WT PAO1 strain; thus, the genetic manipulations are limited and challenging in those isolates. In order to demonstrate the efficiency of our method for a clinical isolate, the 39016 strain of *P. aeruginosa* was tested for curing its second prophage (PR2).

First, 39016/PR2_*ampR* mutant was created by inserting the *ampR* cassette instead of the PA39016_000100015 gene (hypothetical protein, herein termed as AmpRin region). Next, to assure that the insertion did not affect the excision ability of PR2, a comparison of the infectivity of phages produced by WT 39016 or by 39016/PR2_*ampR* strain has been performed. The results showed that the cassette’s insertion did not impair the bacterial growth or the infectious phage production of the 39016 strain (Figure S1).

**Figure 4:**
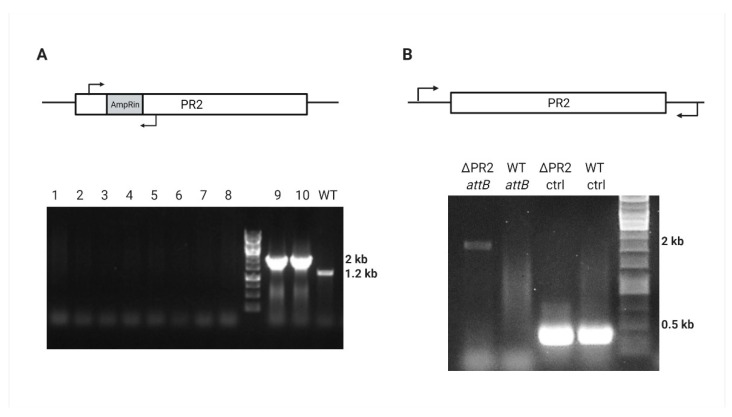
PR2 curing verification by PCR. (**A**) AmpRin region amplification results either in null, 2000 bp, or 1200 bp in the cured, *ampR*-containing, or WT strain, respectively; lanes 1–8 represent randomly picked Crb-sensitive colonies, lanes 9–10 represent Crb-resistant colonies, and the WT 39016 is for positive control. (**B**) Amplification of 2000 bp around the PR2 *attB* site of ΔPR2 and WT 39016, and positive control (ctrl) amplification of 324 bp PA39016_100004 external gene.

Further *sacB* integration was directed by homologous sequences upstream and downstream to the intragenic region of PR2 located between the PA39016_000100034 and PA39016_000100035 genes (SacBin region). After the sucrose counter-selection, the antibiotic screen revealed approximately 70% (57/80) Crb-sensitive colonies that were also Gm resistant. Prophage gene amplification indicated that the phage was lost (Figure 4A); however, the SacBin region was still present in the cured strain (Figure S2) indicating some recombinant plasmid has been created in the process that contains the *accC1* marker and the SacBin region; this strain was named “ΔPR2/Gm^R^.” In order to cure the recombinant plasmid, we applied a temperature-based plasmid curing assay on ΔPR2/Gm^R^ strain. PCR confirmed the resulting Gm-sensitive colonies for the SacBin region loss and *attB* region amplification for the complete phage loss (Figure 4B). One such mutant was chosen for study and designated as ΔPR2 strain.

**Figure 5:**
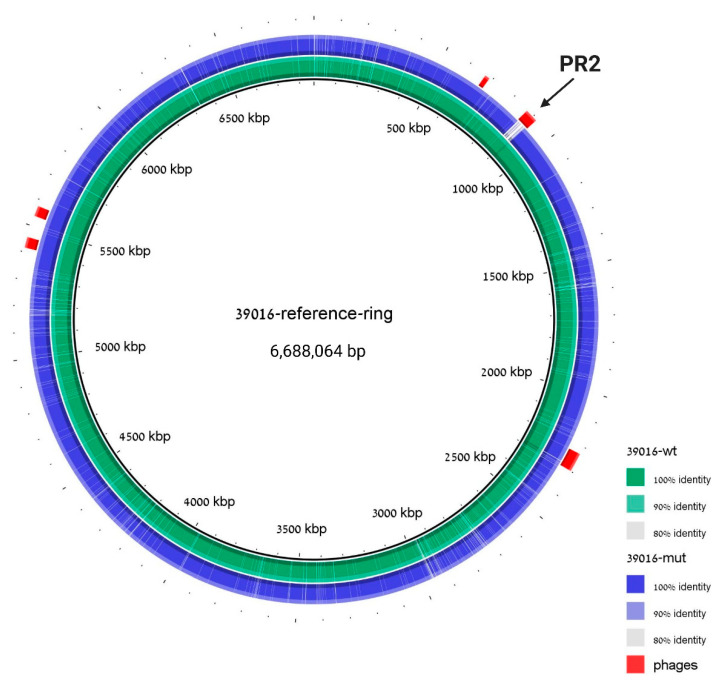
The ΔPR2 strain differs from WT in the PR2 region solely. Genomic alignment with 39016 strain is used as a reference, 39016 WT sequence assembly is indicated in green, and ΔPR2 strain sequence assembly is indicated in blue. Prophage regions are labeled in red; coordinates are taken from PHASTER analysis to the reference 39016 strain. The cured region is marked with an arrow.

To verify that both the prophage-curing and the plasmid-curing processes did not result in a high mutation rate in the ΔPR2 strain, the cured strain genome was sequenced and aligned to the WT 39016 sequence. The genome analysis revealed that PR2 was missing in the cured strain, and no additional significant insertions or deletions were detected (Figure 5). Moreover, PHASTER [30] analysis of the assembled genome of ΔPR2 revealed that the cured strain differs from the WT 39016 in its prophage content solely in the second phage; the curing process did not affect all the other phages of 39016.

Unfortunately, the attempts to use the cured ΔPR2 strain as a host by infecting with phages extracted from WT 39016 strain had failed, suggesting some cross-immunity between PR2 and the other prophages in 39016. However, as indicated by PCR amplification of the extracted intact phages, while successful PR2 virion production was observed in the WT strain, it is not produced by the cured strain (Figure S3).

## 4. Discussion

In this study, we provide a simple counter-selection-based curing methodology. The approach was proven successful for both the laboratory PAO1 WT strain and the clinical, hard to genetically manipulate, 39016 strain. The guiding principles of the targeted method were first to avoid DNA damaging as in the usage of SOS response promoting agents, and second, to cure a specific prophage without influencing the other prophages in the genome of the poly-lysogenic strains.

The strategy for prophage targeted curing from poly-lysogenic strains should take into the similarity of the different prophages. For poly-lysogenic strains that carry different prophages, there is no need for a spatial approach; as we have shown with 39016, the other prophages are not affected by the curing process. For strains that carry highly similar prophages, the tagging step should be directed to a unique region or a low-similarity region within the prophage. PAO1 is an example of such a ploy-lysogenic strain as it carries two highly similar prophages in its genome; Pf4 and Pf6 prophages [32]. We achieved 100% curing by focusing on the unique region of Pf4 for the *ampR* insertion step to assure proper prophage-tagging, and the second step was directed into a common region with high similarity for both Pf6 and Pf4. For strains that carry multiple copies of the same prophage, it should be recommended to sequence properly following the tagging step to identify which of the prophages was eventually targeted to be cured in the second step.

The use of a counter-selection marker for prophage curing has been described before for *Streptococcus pyogenes* [33]. The method is based on first creating a streptomycin-resistant strain that is mutated in its *rpsL* gene, and further insertion of other antibiotic resistance cassette and the *rpsL* WT allele for counter-selection by streptomycin. The difficulty of using such an approach in *P. aeruginosa* is mainly because it does not harbor streptomycin resistance through genomic mutations in the *rpsL* gene [34], so the counter-selection marker is not suitable in this case. Furthermore, the resistant mutant creation process by genomic mutation might disrupt the genomic integrity and cause additional, uncontrolled mutations. Another paper described a different counter-selection-based curing technique in *Vibrio natriegens* by inserting an inducible *ccdB* toxin into the phage region and further counter-selection by toxin induction [34]. The usage of *ccdB* toxin or other toxins from TA modules might be problematic for targeted curing based on the assumption that the toxins might induce other prophages in the genome regardless of the curing process [23]. Our approach avoids such problems.

It is important to note that the curing process of PR2 from 39016 had, in addition to the targeted curing process, an additional plasmid curing step to remove the recombinant plasmid produced in the curing process. The creation of the recombinant plasmid might have occurred due to the conservation of an *oriT*, the *atcc1* cassette, and some specific, important regions of the prophage. A similar case was also reported in the curing of specific prophage in *S. pyogenes*. The produced recombinant plasmid in the reported case was related to the prophages’ cross-regulatory properties. When attempting to cure the same phage, in the background of other cured prophages, the recombinant plasmid was not produced [33].

The curing process of Pf4 from PAO1 was very efficient, with a high yield of cured mutants that can serve as a surrogate for infection from PAO1-produced phages. The high yield might be related to the fact that the first curing step included the deletion of the *pfiT* toxin gene; in other cases, it might be less efficient due to the addiction mediated by the stable toxin that will promote killing in the cured, antitoxin free variants. Thus, as part of the curing assay, it is recommended to initially scan for TA systems in the specific prophage sequence and target the toxin in the *ampR* insertion step.

Using the double-stage approach, we managed to overcome the difficulty with the limited counter-selection available markers for *P. aeruginosa*, when in the first stage, the *sacB* marker was used for plasmid-backbone removal, and then, the same marker was utilized to select for complete phage loss from the curing step. It is important to note that the method can potentially be applied to other mobile genetic elements and stable-plasmids that are commonly associated with bacterial virulence [35,36].

The principle of the method and the high observed efficiency gives a better understanding of prophage–host interactions. The prophage-excision event mostly attributed to the phage disadvantage for staying integrated [37] or regulated by the host for a temporary extra-chromosomal state (active lysogeny) [38], but here, we showed that under certain conditions, when the prophage’s contents endanger the host, it would prefer to lose the whole prophage rather than the specific risk-containing region. This fact emphasizes the need for curing rather than deletion methods for such regions.

## Figures and Tables

**Table 1:** Strains and plasmids used in the study.

Strain or plasmid	Description	Source
*P. aeruginosa* strains		
PA01	WT	[22]
PAO1 ΔPf4	PAO1, ΔPf4	This study
PAO1 Δ*pfiT*	PAO1, ΔPAO729::Crb^r^	[23]
39016	LMG 27,647 *P.* *aeruginosa* (Schoeter 1872) migula 1900 AL	BCCM-biological origin: keratitis patients
39016/PR2_ampR	39016, ΔPA39016_000100015::Crb^r^	This study
30916 ΔPR2/Gm^r^	39016, ΔPR2::Gm^r^	This study
30916 ΔPR2	39016, ΔPR2	This study
*E. coli* strains		
DH5α	F- Φ80lacZΔM15 Δ(lacZYA-argF) U169 recA1 endA1 hsdR17 (rK-, mK+) phoA supE44 λ- thi-1 gyrA96 relA1.	Bio-Lab
S17	*E. coli* S17 thi, pro, hsdR, recA::RP4 -2-Tc::Mu aphA::Tn7, λ-pir, Smr, Tpr	[24]
Plasmids		
pUCP18-Ap	Crb^r^ (for *P. aeruginosa*), Amp^r^ (for E. coli), overexpression plasmid, lacZ promoter	[25]
pDONRPEX18Gm	Gm^r^ and Cm^r^, pEX18Gm containing a HindIII flanked, *attP* cloning site from pDONR201	[21]
pDONER/AmpRin_PR2	Cm^r^ and Gm^r^, PA39016_000100015 upstream and downstream fragments with AmpR cassette inserted into pDONRPEX18Gm by Gateway recombination	This study
pDONER/PR2_SacB	Cm^r^ and Gm^r^, PA39016_000100043 and PA39016_000100035 fragments inserted into pDONRPEX18Gm by Gateway recombination	This study
pDONER/Pf4_SacB	Cm^r^ and Gm^r^, PA0725 upstream and downstream fragments inserted into pDONRPEX18Gm by Gateway recombination	This study

^r^ for resistance.

## Data Availability

Not applicable.

## Supplementary Materials

The following are available online at https://www.mdpi.com/1999-4915/13/2/336/s1, Supplementary methods, Table S1: Primers used in the study, Figure S1: *ampR* insertion into the PR2 prophage did not alter the bacterial growth and PAO1-infectious phages production, Figure S2: The SacBin region presents in PR2-cured colonies. Figure S3: PR2 phages are not produced in the cured strain.
