# Supplementary material for: An Efficient, Counter-Selection-Based Method for Prophage Curing in Pseudomonas aeruginosa Strains
Source: Viruses. 2021 Feb 21;13(2):336. doi: 10.3390/v13020336 (PMC7926659; doi:10.3390/v13020336)
Supplement: Supplementary file 1 [file viruses-13-00336-s001.pdf]

Supplementary materials

Supplementary methods

Inducible phage extraction

LB (2 ml) was inoculated with 39016 strain incubated overnight. On the next day, the bacteria were diluted 1:50 with medium to a final volume of 4 ml and incubated until reaching OD 0.5 at 595nm (~2 hours). For phage induction, 0.4 µg/ml norfloxacin (sigma) antibiotic was added, and the cultures were incubated for 1 hour. Fresh LB was added (1.75 mL), and the cultures were incubated for an additional one hour. Then, 1 ml of bacteria was centrifuged at 14,000g for 2 min, and 900 µl of the supernatant was filtered using a 0.45 µm filter (Whatman). For PCR amplification, the extracted phages were treated with 1 µg/ml DNaseI for 60 min at 37°, followed by inactivation of the enzyme activity by incubation at 65° for 15 min.

Growth curve

LB (2 mL) was inoculated with bacterial strains from frozen stocks and incubated overnight at 37°C with shaking (250 rpm). The culture was diluted to 0.005 OD (595 nm) in fresh media and transferred to a 96-well plate, 200µl in each well. The plates were incubated for 15 hours at 370C with agitation. Optical density measurements at 595 nm were taken every 30 minutes using the Synergy™ 2 Multi-Detection Microplate Reader (BioTek).

Table S1: Primers used in the study

Primer	Sequence
**M13_F**	CCCAGTCACGACGTTGTAAAACG
**M13_R**	AGCGGATAACAATTTCACACAGG
**AmpR_F**	CGCGGAACCCCTATTTGTT
**AmpR_R**	TTACCAATGCTTAATCAGTGAGG
**PR2_500Dn_R**	AAGCTTAGGCGATCCAGGCCGAC
**PR2_500up_F**	GAGCTCTATTTTTATTGCGACGACAGCG
**PR2_AmpRin _Seq_F**	GAGCTCATAGATCACCCCCTTGCTCG
**PR2_AmpRin_Up_F_GWB1**	GGGGACAAGTTTGTACAAAAAAGCAGGCTCACAAGCCATTTGCACCAGCAG
**PR2_AmpRin_Up_R**	AACAAATAGGGGTTCCGCGGATACCATGAAACGAGCAACC
**PR2_AmpRin_Down_F**	CCTCACTGATTAAGCATTGGTAACTACTTGCTGATGCCGATGAA
**PR2_AmpRin_Down_R_GWB2**	GGGGACCACTTTGTACAAGAAAGCTGGGTAGTGACGACGCTGTTCCAGA
**PR2_AmpRin _Seq_R**	GAATTCCGTTGAGGGTATGAAGGTTG
**PR2_SacBin_Up_F_GWB1**	GGGGACAAGTTTGTACAAAAAAGCAGGCTCAAACACGGCGAAGCAGAGCT
**PR2_SacBin_Up_R**	GCGCGCACGTATCAACAGATTCAAGCCGCATCGAGCAAC
**PR2_ SacBin _Down_F**	ATCTGTTGATACGTGCGCGC
**PR2_SacBin_Down_R_GWB2**	GGGGACCACTTTGTACAAGAAAGCTGGGTATAGCTGCGTGTTGGCTTGC
**Pf4_RF_F**	AGCAGCGCGATGAAGCAAT
**Pf4_RF_R**	TAGAGGCCATTTGTGACTGGA
**Pf4_seq_F**	TACGAGGCTGTTGAGGAGTTA
**Pf4_seq_R**	CCGTGCCGAGGTAGTGATGTC
**PR5_seq_F**	TCAAACCATCCAATAGCTGGC
**PR5_seq_R**	GGCGGCAGGCGTATCCTT
**PA39016_100004_F**	ATGGTTAAGAAATTCTCCGAC
**PA39016_100004_R**	TTAGGCGCCCGCCTCTTC

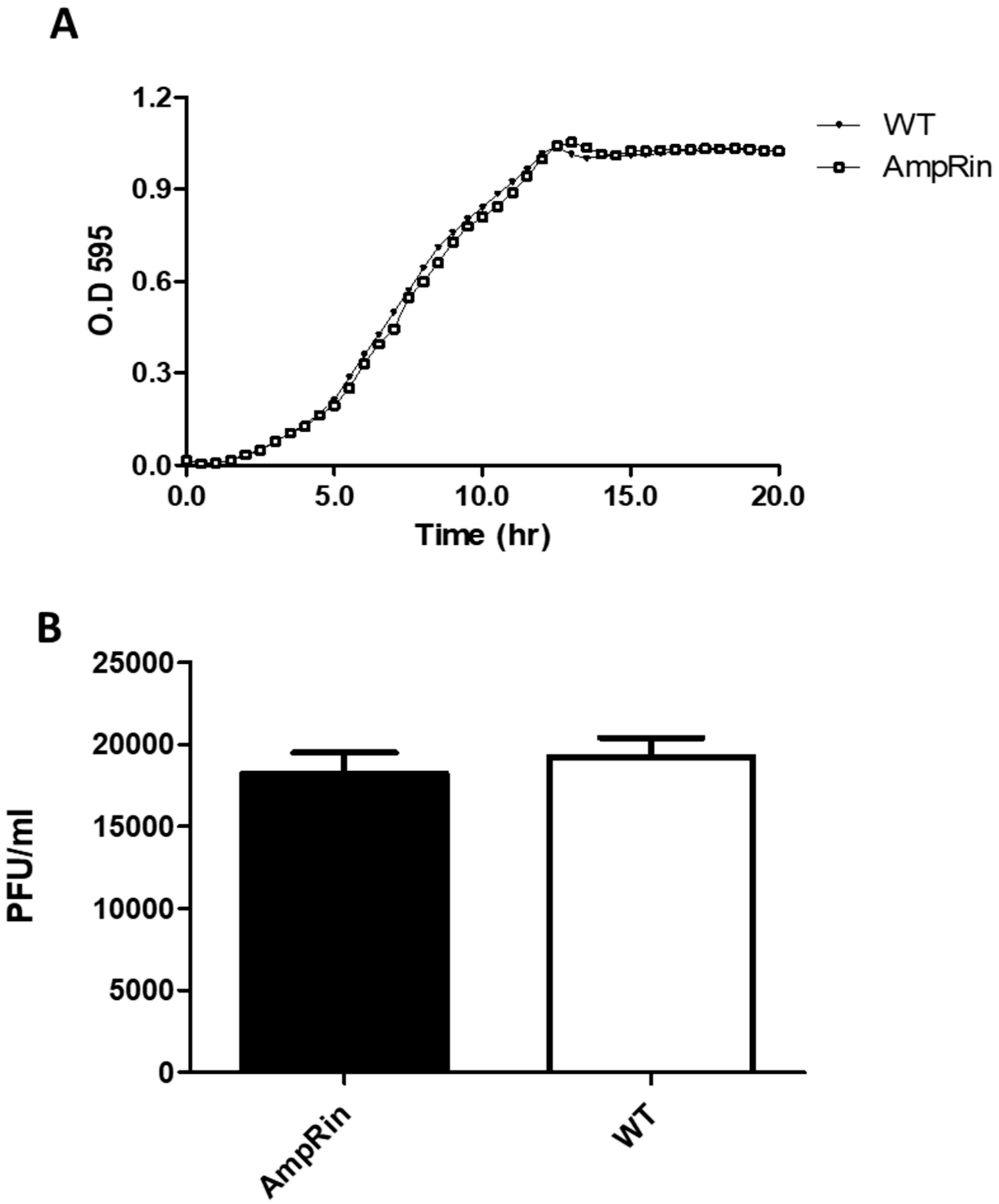

Figure S1: *ampR* insertion into the PR2 prophage did not alter the bacterial growth and PAO1-infectious phages production. (A) The growth curve of *ampR* inserted strain (AmpRin) compared to WT 39016; OD was measured automatically every 30 min. (B) Plaque forming units count, PAO1 was used as a host, phages were induced and extracted from WT, and the *ampR* inserted strain (AmpRin). The above graphs are the average of two independent experiments consisting of (A) five replicates each (B) three replicates each. Error bars represent the standard deviations.

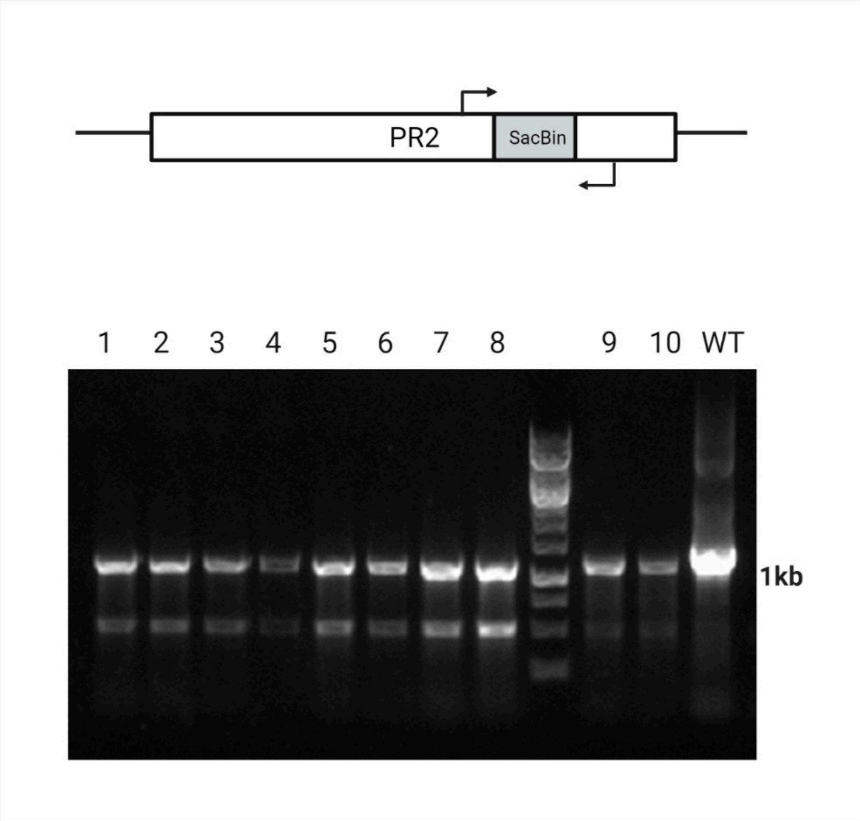

Figure S2: The SacBin region presents in PR2-cured colonies. PCR amplification of 1100bp SacBin region; lanes 1-8 represent randomly picked Crb-sensitive colonies, lanes 9-10 represent Crb-resistant colonies, and the WT 39016 lane for positive control. The *PR2_SacBin_Up_F_GWB1* and *SacBin_Down_R_GWB2* were used for the amplification.

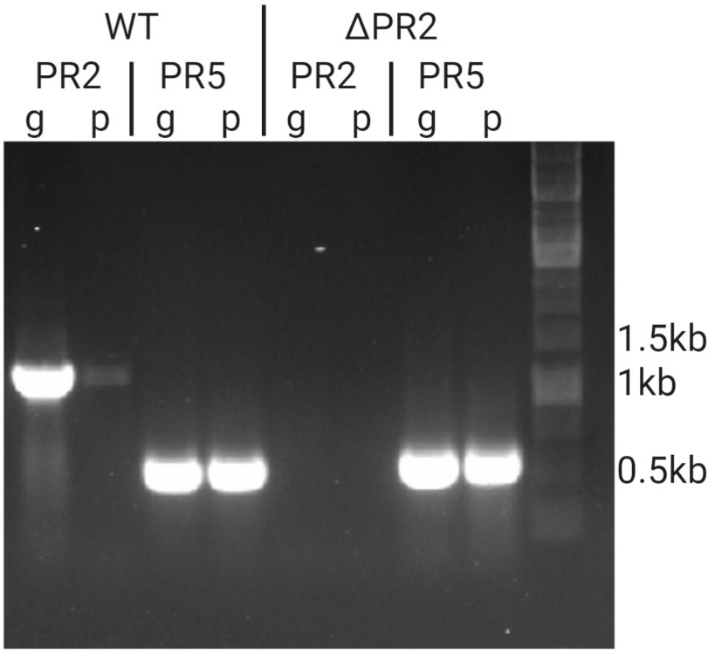

Figure S3: PR2 phages are not produced in the cured strain. PCR amplification of 1200bp region from PR2 phage and 500bp region from a different inducible-prophage (PR5), both for genomic DNA (g) and for phages induced and extracted (p) from 39016 WT and ΔPR2 strains.
